# Supplementary material for: NOG1 increases grain production in rice
Source: Nat Commun. 2017 Nov 14;8:1497. doi: 10.1038/s41467-017-01501-8 (PMC5684330; doi:10.1038/s41467-017-01501-8)
Supplement: Supplementary file 1 — Supplementary Information [file 41467_2017_1501_MOESM1_ESM.pdf]

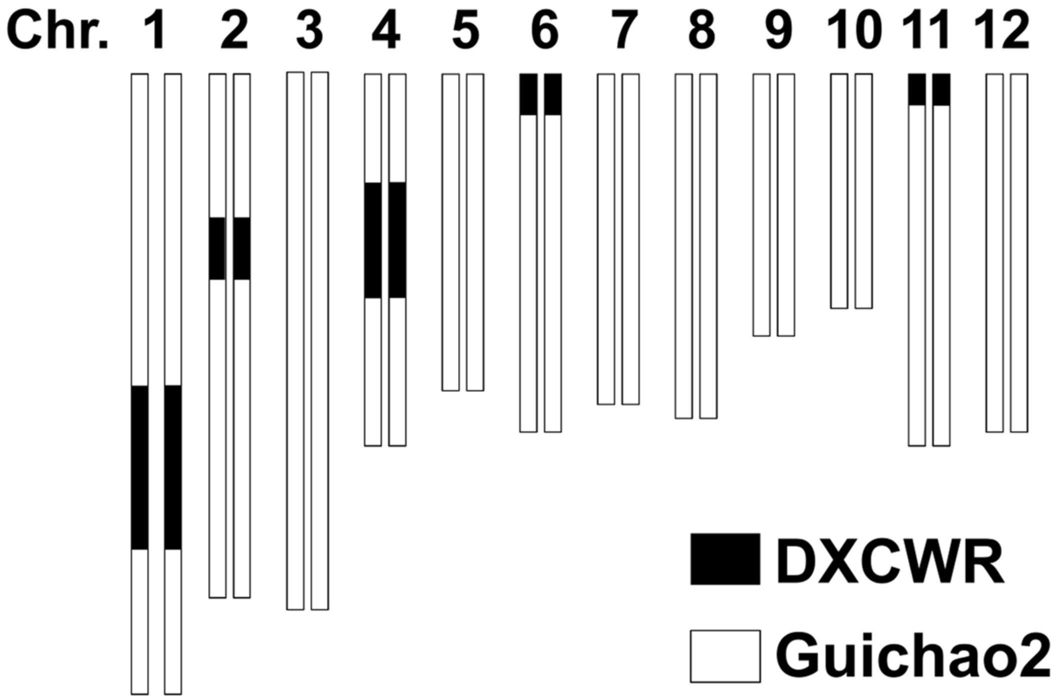

Supplementary Figure 1 | Genome constitution of introgression line SIL176.

The black regions indicate Dongxiang common wild rice (DXCWR) introgressions. The white regions indicate Guichao 2 genetic background.

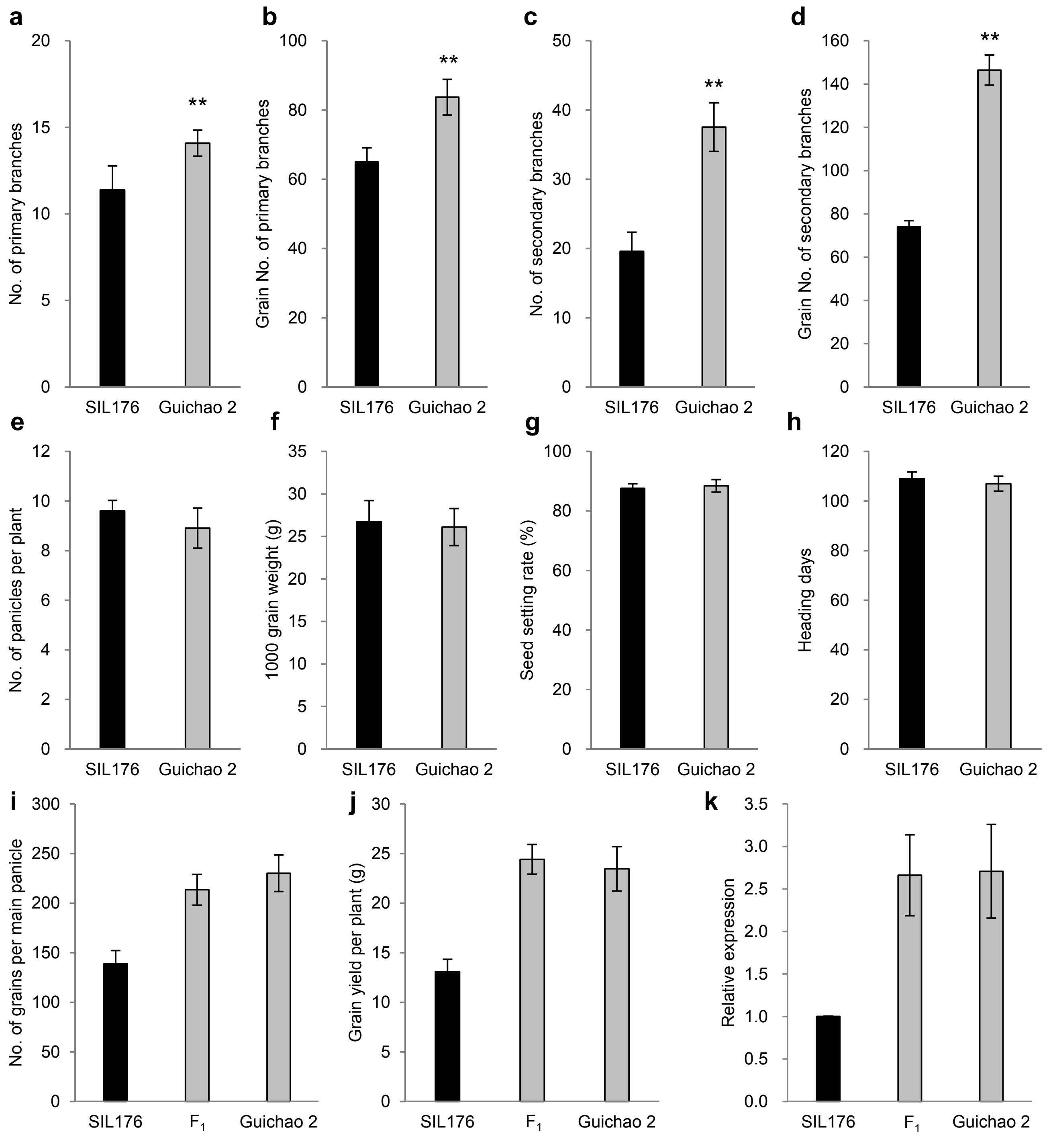

Supplementary Figure 2 | Phenotype of parental and F_1 plants.

(a-d) Comparison of number of primary branches, grain number of primary branches, number of secondary branches, and grain number of secondary branches on the main culm between SIL176 and Guichao 2 (n=30).

(e-h) Comparison of number of panicles per plant, 1000 grain weight, seed setting rate, and heading date between SIL176 and Guichao 2. Data were derived from Beijing, China (latitude 40 degrees north) (n=30).

(i-j) Comparison of number of grains per panicle and grain yield per plant among SIL176, Guichao 2, and F_1 plants (n=10).

(k) Comparison of relative expression levels of *NOG1* among SIL176, Guichao 2, and F_1 plants (n=4).

Data are means, with error bars showing SEM (two-tailed Student's *t* test; **, $P < 0.01$).

a

Guichao 2	CACTTTTGACTCTATATAGTGTGTTCTTAATCGGTGAAACGAAATGTACT	50
SIL176	CACTTTTGACTCTATATAGTGTGTTCTTAATCGGTGAAACGAAATGTACT	50
Guichao 2	AGTCATTTCATGTGCCATACAATGTTATTGCTAATTTTTACACGAATGCT	100
SIL176	AGTCATTTCATGTGCCATACAATGTTATTGCTAATTTTTACACGAATGCT	100
Guichao 2	AGTCTATTTCACTGATGTCTTTTTTTCCATGCTCGGTGTTAGATTTGATT	150
SIL176	AGTCTATTTCACTGATGTCTTTTTTTCCATGCTCGGTGTTAGATTTGATT	150
Guichao 2	GCCTACTGCTAGACTTATTGTCGTTTTTTTTCCTGATGTACAACTGTGTA	200
SIL176	GCCTACTGCTAGACTTATTGTCGTTTTTTTTCCTGATGTACAACTGTGTA	200
Guichao 2	TACTTGTATTATGACTTATTCTGATTCTTCTGCTGTACATTTACCCCTTG	250
SIL176	TACTTGTATTATGACTTATTCTGATTCTTCTGCTGTACATTTACCCCTTG	250
Guichao 2	GAGATTTTATGATAAATTACGATTGTGGATACACAATGATTTAAATTGGC	300
SIL176	GAGATTTTATGATAAATTACGATTGTGGATACACAATGATTTAAATTGGC	300
Guichao 2	TGAGTTAGTTCACGCTGAGTTTATTATCAGCTTTTTCAATAGTTTACCTG	350
SIL176	TGAGTTAGTTCACGCTGAGTTTATTATCAGCTTTTTCAATAGTTTACCTG	350
Guichao 2	ATAATATTTCCTTTTGTTGTTTTATCTGCAGATTTTTGAGAAGAACCCAA	400
SIL176	ATAATATTTCCTTTTGTTGTTTTATCTGCAGATTTTTGAGAAGAACCCAA	400
Guichao 2	CGACTATCAAGAACTATGGCATCTGGCTGCGTTACCAGAGTAGGACTGGC	450
SIL176	CGACTATCAAGAACTATGGCATCTGGCTGCGTTACCAGAGTAGGACTGGC	450
Guichao 2	TACCACAACATGTACAAGGAGTACCGTGATACCACCCTCAATGGTGCCGT	500
SIL176	TACCACAACATGTACAAGGAGTACCGTGATACCACCCTCAATGGTGCCGT	500
Guichao 2	TGAGCAGATGTACACTGAGATGGCTTCCCGCCACCGTGTCAGATTCCCTT	550
SIL176	TGAGCAGATGTACACTGAGATGGCTTCCCGCCACCGTGTCAGATTCCCTT	550
Guichao 2	GCATTCAGATCATCAAGACTGCTACTGTCCACTTTAAACTCTGCAAGCGT	600
SIL176	GCATTCAGATCATCAAGACTGCTACTGTCCACTTTAAACTCTGCAAGCGT	600
Guichao 2	GACAACACTAAGCAGTTCCACAAGTCAGATATCAAGTTCCCACTGGTGTA	650
SIL176	GACAACACTAAGCAGTTCCACAAGTCAGATATCAAGTTCCCACTGGTGTA	650

Guichao 2	C A G G A A G G T C A G G C C C C C T A C C A G G A A G C T G A A G A C A A C C T T C A A A G C T T	700
SIL176	C A G G A A G G T C A G G C C C C C T A C C A G G A A G C T G A A G A C A A C C T T C A A A G C T T	700
Guichao 2	C A A G G C C A A A C T T G T T T A T G T G A T C C A A G T C G C C T T C C A T G T T C T G T A A G	750
SIL176	C A A G G C C A A A C T T G T T T A T G T G A T C C A A G T C G C C T T C C A T G T T C T G T A A G	750
Guichao 2	A T C T G C C A G T A T G G T G T A G T C G T A G T T C A G A A C T T A A G A T G A A T C T T A T C	800
SIL176	A T C T G C C A G T A T G G T G T A G T C G T A G T T C A G A A C T T A A G A T G A A T C T T A T C	800
Guichao 2	C A T A T G A A G C C T G T T T T T G A A A A G T T C T G T T A G G T C C A T G C T G C G G T C T C	850
SIL176	C A T A T G A A G C C T G T T T T T G A A A A G T T C T G T T A G G T C C A T G C T G C G G T C T C	850
Guichao 2	T C T A G G T T C T C G T A T C G T A T C C T C G T A T C C T T T T C T T T T G C T T T G T G A T C	900
SIL176	T C T A G G T T C T C G T A T C G T A T C C T C G T A T C C T T T T C T T T T G C T T T G T G A T C	900
Guichao 2	A T C T G A T G C C T C A T A C T G A C A A T T C C C A G T T T G C G C T G A T A T T G G T T T A C	950
SIL176	A T C T G A T G C C T C A T A C T G A C A A T T C C C A G T T T G C G C T G A T A T T G G T T T A C	950
Guichao 2	T G A A A T A T T A T A A C G T T G C T A T T T T G C T C A C T G C T T A T G G T A G C C C A A T T	1000
SIL176	T G A A A T A T T A T A A C G T T G C T A T T T T G C T C A C T G C T T A T G G T A G C C C A A T T	1000
Guichao 2	G A A T A A T C C G T A A A A T C A T G G T C T G A T T G A A C T G T G T T T C A A T T T T C T T G	1050
SIL176	G A A T A A T C C G T A A A A T C A T G G T C T G A T T G A A C T G T G T T T C A A T T T T C T T G	1050
Guichao 2	C T G T A T A T G C T A A T C T T A G T G A C T T G C A T C A A T T G G G T G T T A T G T G C T T A	1100
SIL176	C T G T A T A T G C T A A T C T T A G T G A C T T G C A T C A A T T G G G T G T T A T G T G C T T A	1100
Guichao 2	T T A C G T A A T T C T G T A A A A C G A A A A A T A C A A G T T A A T T C T T C A T G A T T G T T	1150
SIL176	T T A C G T A A T T C T G T A A A A C G A A A A A T A C A A G T T A A T T C T T C A T G A T T G T T	1150
Guichao 2	T T T T G T T T C A T G C T T T C A T A T T T C A T T G G T T A T T T G T T T G G C A C A A C C G C	1200
SIL176	T T T T G T T T C A T G C T T T C A T A T T T C A T T G G T T A T T T G T T T G G C A C A A C C G C	1200
Guichao 2	A C A A A G C T T T A G C T A T C T A T T T T G G C T G A T A T T G G G T A T G C A T C A T T T T A	1250
SIL176	A C A A A G C T T T A G C T A T C T A T T T T G G C T G A T A T T G G G T A T G C A T C A T T T T A	1250
Guichao 2	C T G A T A G G G A T A T T G A T T G A T G C T G G A T A A A A A T G T T T T T C T T G C A T C A C	1300
SIL176	C T G A T A G G G A T A T T G A T T G A T G C T G G A T A A A A A T G T T T T T C T T G C A T C A C	1300

12-bp InDel -2214

```
Guichao 2  ATTCAAACTTTTGAGTAGTTAGGATGGCAACTTTGATGGCAACTTTGCCG  1350
SIL176     ATTCAAACTTTTGAGTAGTTAGGATGGCAACTTTG------------CCG  1338
```

-2166

```
Guichao 2  AGGTTACCAACAGTTAATTTGGTTCCCTTTTTTTCCCTGTTGCCTATATC  1400
SIL176     AGGTTACCAACAGTTAATTTGGTTCCCTTTTTTTCCCTGTTGCCAATATC  1388

Guichao 2  TGATTAACTCTTGGGTGCTCTCTTTTTTCCATAAACCTAGTGGTTTATTG  1450
SIL176     TGATTAACTCTTGGGTGCTCTCTTTTTTCCATAAACCTAGTGGTTTATTG  1438

Guichao 2  GATAAGCATCCTAGCTAACATCAGCTGCTAAAATAAAGTAGTTTTACTTT  1500
SIL176     GATAAGCATCCTAGCTAACATCAGCTGCTAAAATAAAGTAGTTTTACTTT  1488

Guichao 2  TTAAGTTCTCTTACTAGTCGCATTGTGAGGTTTTGTTAGGCAACTTTGCC  1550
SIL176     TTAAGTTCTCTTACTAGTCGCATTGTGAGGTTTTGTTAGGCAACTTTGCC  1538

Guichao 2  GAGGTTACCAACAGTTAATTTGGTTACCTTTTTTTCCCTGTTGCCAATAT  1600
SIL176     GAGGTTACCAACAGTTAATTTGGTTACCTTTTTTTCCCTGTTGCCAATAT  1588

Guichao 2  CTGATTAGCTGTTAGGTGCTATTCTTTCCATAAACCTAGTGGTTTAATGG  1650
SIL176     CTGATTAGCTGTTAGGTGCTATTCTTTCCATAAACCTAGTGGTTTAATGG  1638

Guichao 2  ATGAGCATCTTAGCTAACATCAGCTGCTAAAATAAATAACGATGTACTTT  1700
SIL176     ATGAGCATCTTAGCTAACATCAGCTGCTAAAATAAATAACGATGTACTTT  1688

Guichao 2  TAAGTTCTGATCATTCTTTCGTTTTGTTTTCATAATCTAATTCAATGTTC  1750
SIL176     TAAGTTCTGATCATTCTTTCGTTTTGTTTTCATAATCTAATTCAATGTTC  1738
```

-1764

```
Guichao 2  TGTGACTCCATAAATCATTTTTTTTACTTGGGCTTTATATCTCTTCGTAA  1800
SIL176     TGTGACTCCATAAATCATTTTTTTTACTTGGGCTTTATATCTCTTCCTAA  1788

Guichao 2  TGCAAGACGGTATGCAAATTTTGGTCATTTCAATTATGTACAAACTCATC  1850
SIL176     TGCAAGACGGTATGCAAATTTTGGTCATTTCAATTATGTACAAACTCATC  1838

Guichao 2  CCAGTTGCATTTGCTACCTGTGAAGTTGTTAGGCAACTTTGCCGAGGTTA  1900
SIL176     CCAGTTGCATTTGCTACCTGTGAAGTTGTTAGGCAACTTTGCCGAGGTTA  1888

Guichao 2  CCAAGACATGTTTGGTTTCCTTTTTTTCCCTGTTGCCTATATCATTAGTT  1950
SIL176     CCAAGACATGTTTGGTTTCCTTTTTTTCCCTGTTGCCTATATCATTAGTT  1938
```

Guichao 2	GCTTGGTTGCTTGAACTCCATTGACCTATTTGGTTTTTTGGGTATGCATC	2000
SIL176	GCTTGGTTGCTTGAACTCCATTGACCTATTTGGTTTTTTGGGTATGCATC	1988
Guichao 2	CATGCCAACATATAATATTTTCCATCATTCTTTTTACACTTAGCACTGCC	2050
SIL176	CATGCCAACATATAATATTTTCCATCATTCTTTTTACACTTAGCACTGCC	2038
Guichao 2	CAAATGTTGGTTTGGTTCGTGGACTGAGATTCTCGGGAGGCTTTTATGTT	2100
SIL176	CAAATGTTGGTTTGGTTCGTGGACTGAGATTCTCGGGAGGCTTTTATGTT	2088
Guichao 2	TTTTGATCGAATGGTTCGTGGTTTCTTTGCTGGTTTGTGGTTATTTTATC	2150
SIL176	TTTTGATCGAATGGTTCGTGGTTTCTTTGCTGGTTTGTGGTTATTTTATC	2138
Guichao 2	TTCTTGGAGTATATGTTTGTCGAGATAATTGTGGATGTAAATGCAGTCGT	2200
SIL176	TTCTTGGAGTATATGTTTGTCGAGATAATTGTGGATGTAAATGCAGTCGT	2188
Guichao 2	AAAGCTCTTAAACGAACTCTACCAACAGCTCTTATTCCGAAAAAGTAGCT	2250
SIL176	AAAGCTCTTAAACGAACTCTACCAACAGCTCTTATTCCGAAAAAGTAGCT	2238
Guichao 2	AAACTGAAGGATGAAGAACTCTGGTTGGGCGGCCGAGCACCTGATTATCG	2300
SIL176	AAACTGAAGGATGAAGAACTCTGGTTGGGCGGCCGAGCACCTGATTATCG	2288
	-1238	
Guichao 2	GCCACTGCCTTACGAGTACTTTCCGTCTGTGAGGTCGACTCGACATCAAC	2350
SIL176	GCCACTGCCTTACGAGTACTTTTCGTCTGTGAGGTCGACTCGACATCAAC	2338
Guichao 2	CCTTGGAATTCCGTCTGTGTGGTACACAGGATTTGTTATTTAGTAATTGT	2400
SIL176	CCTTGGAATTCCGTCTGTGTGGTACACAGGATTTGTTATTTAGTAATTGT	2388
	-1157 -1141	
Guichao 2	AACCATTCAAATTATCTACTGCTATTCATTTCAAGTTTTCAACTTACCTA	2450
SIL176	AACTATTCAAATTATCTACCGCTATTCATTTCAAGTTTTCAACTTACCTA	2438
	-1099	
Guichao 2	GAAGTGTTTTTAGTCGCTCGTCAATCGTCATGATAACGAATGCTCATACA	2500
SIL176	GAAGTGTTTTTTGTCGCTCGTCAATCGTCATGATAACGAATGCTCATACA	2488
Guichao 2	ACAAACTGAACCAGACGATACGGACGAAACTGCATGAAGCTAGAATAGCT	2550
SIL176	ACAAACTGAACCAGACGATACGGACGAAACTGCATGAAGCTAGAATAGCT	2538
Guichao 2	GTGTGTTCTAGCAGTGTTGTCTCGTAGTAATACTGTACGTACCAGATCGT	2600
SIL176	GTGTGTTCTAGCAGTGTTGTCTCGTAGTAATACTGTACGTACCAGATCGT	2588

```
                                                    -925
Guichao 2  CCAGTACATTCAAATTTTTCACCAAGTCAAGAACTGAGTTCTGGAATCGT  2650
SIL176     CCAGTACATTCAAATTTTTCACCAAGTCAAGAACTAAGTTCTGGAATCGT  2638

Guichao 2  TAAGTGAAGAACTGAGTTCTCGAATCGTGTGTGATTTGTATAGTTCCGAG  2700
SIL176     TAAGTGAAGAACTGAGTTCTCGAATCGTGTGTGATTTGTATAGTTCCGAG  2688

Guichao 2  ATTAGTACTCCCTCCGATTAAGGTTTTAATACGTTGTTAGTAAAAAAAAG  2750
SIL176     ATTAGTACTCCCTCCGATTAAGGTTTTAATACGTTGTTAGTAAAAAAAAG  2738

                                     -786
Guichao 2  GTTTTAATACGTTTTGATTTTAGTCAAAGTTAAAGTTAAACTGTTTTAAG  2800
SIL176     GTTTTAATACGTTTTGATTTTAGTAAAAGTTAAAGTTAAACTGTTTTAAG  2788

                             -742
Guichao 2  TTTGACTAAATTTATAGACAAATATAATAATATTTATAATACTAAATTAG  2850
SIL176     TTTGACTAAATTTATAGATAAATATAATAATATTTATAATACTAAATTAG  2838

Guichao 2  TTTTATCAAATCAATAATTAAATATATTTTCATAATAAATTTATTAATGG  2900
SIL176     TTTTATCAAATCAATAATTAAATATATTTTCATAATAAATTTATTAATGG  2888

Guichao 2  AATATATTTTCATAATAAATTGTTTGGGGTTAAAAATGTTACTATTTTTT  2950
SIL176     AATATATTTTCATAATAAATTGTTTGGGGTTAAAAATGTTACTATTTTTT  2938

Guichao 2  TATACAAACTTGGTTAAACTTAAATCAGTTTGACTTTGACTAAACTCAAA  3000
SIL176     TATACAAACTTGGTTAAACTTAAATCAGTTTGACTTTGACTAAACTCAAA  2988

           -559 -556
Guichao 2  ATGTTTTATAATCTAAAACAGAGGGAGTAGTAATATATACTCTCTCTGTC  3050
SIL176     ACGTCTTATAATCTAAAACAGAGGGAGTAGTAATATATACTCTCTCTGTC  3038

                              -491 -489
Guichao 2  TCATTTTAAGTGCAACTATGATTTTCCGTATCCAACGTTGATTTTCCGTT  3100
SIL176     TCATTTTAAGTGCAACTATAAGTTTCCGTATCCAACGTTGATTTTCCGTT  3088

                                                       -421
Guichao 2  TTATTTGAAAATTTTTTATAATTAGTATTTTTTATTGTTATGAGATGATAA  3150
SIL176     TTATTTGAAAATTTTTTATAATTAGTATTTTTTATTGTTAAGAGATGATAA  3138

                -405
Guichao 2  AATATAAATAGTAATTTATGCGTGACTTATATTTTAGCTTTTTTTTCAAA  3200
SIL176     AATATGAATAGTAATTTATGCGTGACTTATATTTTAGCTTTTTTTTCAAA  3188

Guichao 2  TAAGACGAAAAATCGTAGTCACACTTAAAATGGAAATGGGGCGGCGGAGG  3250
SIL176     TAAGACGAAAAATCGTAGTCACACTTAAAATGGAAATGGGGCGGCGGAGG  3238
```

```
Guichao 2  GAGGACTAGTCCACTGATAAGTGATAACGCATCATTCAAAATGATCCCGA  3300
   SIL176  GAGGACTAGTCCACTGATAAGTGATAACGCATCATTCAAAATGATCCCGA  3288

Guichao 2  AGTGAAAACCGATAAATTCTCAAAGAGGTGTGAATTAGAAGTCAAACTGC  3350
   SIL176  AGTGAAAACCGATAAATTCTCAAAGAGGTGTGAATTAGAAGTCAAACTGC  3338

Guichao 2  ATCGACCCAGCTGTCCATGGTCTTTTTCCGTTCGCCCTCGCTTTCTTCCC  3400
   SIL176  ATCGACCCAGCTGTCCATGGTCTTTTTCCGTTCGCCCTCGCTTTCTTCCC  3388

Guichao 2  GCACCAACCCCGCCTACCGCTCCACCACTACTACTACACCATCCCCTCCA  3450
   SIL176  GCACCAACCCCGCCTACCGCTCCACCACTACTACTACACCATCCCCTCCA  3438

Guichao 2  CCGGCTCCCCCTCACCAAACTTCCCTCCACGCCTGCTTCGCCCAACTCGT  3500
   SIL176  CCGGCTCCCCCTCACCAAACTTCCCTCCACGCCTGCTTCGCCCAACTCGT  3488

Guichao 2  TCTCTTGGAGCACCTAACTCGAGCTGAGCTCCCTTCCCGCGGAATTCGGG  3550
   SIL176  TCTCTTGGAGCACCTAACTCGAGCTGAGCTCCCTTCCCGCGGAATTCGGG  3538

                     +1 TSS
Guichao 2  TCCCTCCCTGATGGCGGAGCCGGAGCAGCAGCAGCAGCAGGCGAATCCCG  3600
   SIL176  TCCCTCCCTGATGGCGGAGCCGGAGCAGCAGCAGCAGCAGGCGAATCCCG  3588

Guichao 2  ACGAGGTACCGATCAGCCCCACGCGAACGAGGCCATTTTCTTCTTTCTTG  3650
   SIL176  ACGAGGTACCGATCAGCCCCACGCGAACGAGGCCATTTTCTTCTTTCTTG  3638

Guichao 2  TAGTATGTGGTGTGGTGAGAGTGAGAACGCGCGGCGCCGTTTCTTTGTGC  3700
   SIL176  TAGTATGTGGTGTGGTGAGAGTGAGAACGCGCGGCGCCGTTTCTTTGTGC  3688

Guichao 2  AGGTGGTGCTCGGGCAGGAGACAGGCGGCGCGAGGGTGGCGATCCTCAAC  3750
   SIL176  AGGTGGTGCTCGGGCAGGAGACAGGCGGCGCGAGGGTGGCGATCCTCAAC  3738

Guichao 2  CGGCCGCGCCAGCTGAACGTCATCTCCGATAGAGTGGTGCGATTTCTTTC  3800
   SIL176  CGGCCGCGCCAGCTGAACGTCATCTCCGATAGAGTGGTGCGATTTCTTTC  3788

Guichao 2  GCTTCGTGCATTTTCCGATCTTATGCCGGAAGCAGCAGCTGGAAGCTGTA  3850
   SIL176  GCTTCGTGCATTTTCCGATCTTATGCCGGAAGCAGCAGCTGGAAGCTGTA  3838

Guichao 2  ATTGGCTGGGCCGTGCTCGGTTTCTGGTTGCAGGTGTATCTCCTCGCCCA  3900
   SIL176  ATTGGCTGGGCCGTGCTCGGTTTCTGGTTGCAGGTGTATCTCCTCGCCCA  3888
```

Guichao 2	GTTCTTGGAGAGCTGGGAGAAAGATGAGGATGCCAAGCTGGTCATCTTCA	3950
SIL176	GTTCTTGGAGAGCTGGGAGAAAGATGAGGATGCCAAGCTGGTCATCTTCA	3938
Guichao 2	AGGTGCGCGCACTGCTGCCCTCTTAGCACCCTGCATTGATAAAGCTCTAG	4000
SIL176	AGGTGCGCGCACTGCTGCCCTCTTAGCACCCTGCATTGATAAAGCTCTAG	3988
Guichao 2	GGCAGAACATAACTGATTATAATTCAGACAAGGATAGGTTTTAGCCTAGT	4050
SIL176	GGCAGAACATAACTGATTATAATTCAGACAAGGATAGGTTTTAGCCTAGT	4038
Guichao 2	GATGTCATGCGTGATTTGATACCAGGAGACAGCTCAAACAAATCATTTAC	4100
SIL176	GATGTCATGCGTGATTTGATACCAGGAGACAGCTCAAACAAATCATTTAC	4088
Guichao 2	CTTACTCTAGTTTGTTTCTTCAAAGTCCTTGCAATGCATGGAGCACTTGG	4150
SIL176	CTTACTCTAGTTTGTTTCTTCAAAGTCCTTGCAATGCATGGAGCACTTGG	4138
Guichao 2	CTAGTAGTACAAACTATGTAGAGAAACGTGTTGCTTGTTTAAGGATTCGG	4200
SIL176	CTAGTAGTACAAACTATGTAGAGAAACGTGTTGCTTGTTTAAGGATTCGG	4188
Guichao 2	TAAGGTGTGCCTGGTCAACATTTGAACTTTACTCAGTAGTTAGTACTGAT	4250
SIL176	TAAGGTGTGCCTGGTCAACATTTGAACTTTACTCAGTAGTTAGTACTGAT	4238
Guichao 2	ACATAAGGTAACGTTTTGATATGGATAAGTTTGGCAATAAGACAGCGATG	4300
SIL176	ACATAAGGTAACGTTTTGATATGGATAAGTTTGGCAATAAGACAGCGATG	4288
Guichao 2	AAATGAGGCGTATCATGTCACAAATGAAACTTTTTGTTATGACTTTGTAT	4350
SIL176	AAATGAGGCGTATCATGTCACAAATGAAACTTTTTGTTATGACTTTGTAT	4338
Guichao 2	GAT	4353
SIL176	GAT	4341

b

1	gaccaaccccgcctaccgctccaccactactactacaccatcccctccaccggctccccc
61	tcaccaaacttccctccacgcctgcttcgcccaactcgttctcttggagcacctaactcg
121	agctgagctcccttcccgcggaattcgggtccctccctgATGGCGGAGCCGGAGCAGCAG
1	M A E P E Q Q
181	CAGCAGCAGGCGAATCCCGACGAGGTGGTGCTCGGGCAGGAGACAGGCGGCGCGAGGGTG
21	Q Q Q A N P D E V V L G Q E T G G A R V
241	GCGATCCTCAACCGGCCGCGCCAGCTGAACGTCATCTCCGATAGAGTGGTGTATCTCCTC
41	A I L N R P R Q L N V I S D R V V Y L L
301	GCCCAGTTCTTGGAGAGCTGGGAGAAAGATGAGGATGCCAAGCTGGTCATCTTCAAGGGG
61	A Q F L E S W E K D E D A K L V I F K G

361	GCTGGACGTGCATTTTCCGCTGGTGGGGATCTAAAGATGTTCTATGAAGGAAAATCAGAT
81	A G R A F S A G G D L K M F Y E G K S D
421	GACTCCTGTCTCGAGGTTGTTTACAGGATGTATTGGCTTTGCTACCATATCCACACGTAT
101	D S C L E V V Y R M Y W L C Y H I H T Y
481	AAGAAAACCGCGGTGGCTCTTGTTAATGGACTTGTCATGGGTGGTGGTGCAGCCATGGTT
121	K K T A V A L V N G L V M G G G A A M V
541	GCTCCACTGAAGTTTGCAGTTGTCACAGAGAAAACAGTCTTCGCAACCCCTGAGGCTAGT
141	A P L K F A V V T E K T V F A T P E A S
601	GTTGGATTACACACAGACTGCAGCTTTTCTTATATCCATTCTAGACTCCCTGGATATTTA
161	V G L H T D C S F S Y I H S R L P G Y L
661	GGGGAGTACCTGGCTTTGACCGGTGCAAGGTTGAATGCAAAGGAAATGATTGCTGCCGGT
181	G E Y L A L T G A R L N A K E M I A A G
721	CTTGCTACTCATTTTGTTCCTTCTGAAAAATTGGAAGAACTTGAAAAATGCCTGCTGAAT
201	L A T H F V P S E K L E E L E K C L L N
781	TTAAACACAGGAGATGAGTCTGCTGTTCGAGCTGCTATTGAAGAGTTCTCAACTGATGTT
221	L N T G D E S A V R A A I E E F S T D V
841	CAACCTGATGAAGATAGTATTTTAAACAAGCTCCCAACTATCAACAAATGTTTCTCTGCT
241	Q P D E D S I L N K L P T I N K C F S A
901	GAGACTATCGAGGACATCATAAAAGCTTTTGAATCAGAAGGGAGCATTGATGGAAACCAA
261	E T I E D I I K A F E S E G S I D G N Q
961	TGGATCGCTACAGTACTGAAGGGCATGCGAAGATCATCTCCTACTTCACTGAAGATGACT
281	W I A T V L K G M R R S S P T S L K M T
1021	CTTCGATCGATCAGAGAAGGTCGGAAGCAGAGCCTGCCGGAATGTTTGAAGAAGGAATTC
301	L R S I R E G R K Q S L P E C L K K E F
1081	CGACTTACAATGAACACTCTCCGATCTGTAGTTACTGGCGATGTCTATGAGGGAATTAGA
321	R L T M N T L R S V V T G D V Y E G I R
1141	GCTCTCAGCATCGACAAAGACAATGCCCCTAAGTGGAGTCCTGCTACCCTTGAGGTCAAG
341	A L S I D K D N A P K W S P A T L E V K
1201	AACGAGGACATCGACCGTCTTTTCGAACCATTCAGTTCAGAAAAGGAGCTCCAAGTCCCA
361	N E D I D R L F E P F S S E K E L Q V P
1261	TCTGACGATTCCAACAGATGGAGTGGCAAATTTGAGCACACAGTCTATGGCAGAACTTCA
381	S D D S N R W S G K F E H T V Y G R T S
1321	GAGTAAttggcagcagaaatagatgttcatcatcctcacaagcatgttttaagtactcgt
401	E *
1381	aaccccatgtattctcatacttccctgtataacattgtaacacaaaaaattgaaaattac
1441	atgacgcacgaagcaataaagcatctcaaatgttttgtt

c

Guichao 2	GACCAACCCCGCCTACCGCTCCACCACTACTACTACACCATCCCCTCCAC	50
SIL176	GACCAACCCCGCCTACCGCTCCACCACTACTACTACACCATCCCCTCCAC	50
Guichao 2	CGGCTCCCCCTCACCAAACTTCCCTCCACGCCTGCTTCGCCCAACTCGTT	100
SIL176	CGGCTCCCCCTCACCAAACTTCCCTCCACGCCTGCTTCGCCCAACTCGTT	100

Guichao 2 C T C T T G G A G C A C C T A A C T C G A G C T G A G C T C C C T T C C C G C G G A A T T C G G G T 150
SIL176 C T C T T G G A G C A C C T A A C T C G A G C T G A G C T C C C T T C C C G C G G A A T T C G G G T 150

Guichao 2 C C C T C C C T G A T G G C G G A G C C G G A G C A G C A G C A G C A G C A G G C G A A T C C C G A 200
SIL176 C C C T C C C T G A T G G C G G A G C C G G A G C A G C A G C A G C A G C A G G C G A A T C C C G A 200

Guichao 2 C G A G G T G G T G C T C G G G C A G G A G A C A G G C G G C G C G A G G G T G G C G A T C C T C A 250
SIL176 C G A G G T G G T G C T C G G G C A G G A G A C A G G C G G C G C G A G G G T G G C G A T C C T C A 250

Guichao 2 A C C G G C C G C G C C A G C T G A A C G T C A T C T C C G A T A G A G T G G T G T A T C T C C T C 300
SIL176 A C C G G C C G C G C C A G C T G A A C G T C A T C T C C G A T A G A G T G G T G T A T C T C C T C 300

Guichao 2 G C C C A G T T C T T G G A G A G C T G G G A G A A A G A T G A G G A T G C C A A G C T G G T C A T 350
SIL176 G C C C A G T T C T T G G A G A G C T G G G A G A A A G A T G A G G A T G C C A A G C T G G T C A T 350

Guichao 2 C T T C A A G G G G G C T G G A C G T G C A T T T T C C G C T G G T G G G G A T C T A A A G A T G T 400
SIL176 C T T C A A G G G G G C T G G A C G T G C A T T T T C C G C T G G T G G G G A T C T A A A G A T G T 400

Guichao 2 T C T A T G A A G G A A A A T C A G A T G A C T C C T G T C T C G A G G T T G T T T A C A G G A T G 450
SIL176 T C T A T G A A G G A A A A T C A G A T G A C T C C T G T C T C G A G G T T G T T T A C A G G A T G 450

Guichao 2 T A T T G G C T T T G C T A C C A T A T C C A C A C G T A T A A G A A A A C C G C G G T G G C T C T 500
SIL176 T A T T G G C T T T G C T A C C A T A T C C A C A C G T A T A A G A A A A C C G C G G T G G C T C T 500

Guichao 2 T G T T A A T G G A C T T G T C A T G G G T G G T G G T G C A G C C A T G G T T G C T C C A C T G A 550
SIL176 T G T T A A T G G A C T T G T C A T G G G T G G T G G T G C A G C C A T G G T T G C T C C A C T G A 550

Guichao 2 A G T T T G C A G T T G T C A C A G A G A A A A C A G T C T T C G C A A C C C C T G A G G C T A G T 600
SIL176 A G T T T G C A G T T G T C A C A G A G A A A A C A G T C T T C G C A A C C C C T G A G G C T A G T 600

Guichao 2 G T T G G A T T A C A C A C A G A C T G C A G C T T T T C T T A T A T C C A T T C T A G A C T C C C 650
SIL176 G T T G G A T T A C A C A C A G A C T G C A G C T T T T C T T A T A T C C A T T C T A G A C T C C C 650

Guichao 2 T G G A T A T T T A G G G G A G T A C C T G G C T T T G A C C G G T G C A A G G T T G A A T G C A A 700
SIL176 T G G A T A T T T A G G G G A G T A C C T G G C T T T G A C C G G T G C A A G G T T G A A T G C A A 700

Guichao 2 A G G A A A T G A T T G C T G C C G G T C T T G C T A C T C A T T T T G T T C C T T C T G A A A A A 750
SIL176 A G G A A A T G A T T G C T G C C G G T C T T G C T A C T C A T T T T G T T C C T T C T G A A A A A 750

Guichao 2	TTGGAAGAACTTGAAAAATGCCTGCTGAATTTAAACACAGGAGATGAGTC	800
SIL176	TTGGAAGAACTTGAAAAATGCCTGCTGAATTTAAACACAGGAGATGAGTC	800
Guichao 2	TGCTGTTCGAGCTGCTATTGAAGAGTTCTCAACTGATGTTCAACCTGATG	850
SIL176	TGCTGTTCGAGCTGCTATTGAAGAGTTCTCAACTGATGTTCAACCTGATG	850
Guichao 2	AAGATAGTATTTTAAACAAGCTCCCAACTATCAACAAATGTTTCTCTGCT	900
SIL176	AAGATAGTATTTTAAACAAGCTCCCAACTATCAACAAATGTTTCTCTGCT	900
Guichao 2	GAGACTATCGAGGACATCATAAAAGCTTTTGAATCAGAAGGGAGCATTGA	950
SIL176	GAGACTATCGAGGACATCATAAAAGCTTTTGAATCAGAAGGGAGCATTGA	950
Guichao 2	TGGAAACCAATGGATCGCTACAGTACTGAAGGGCATGCGAAGATCATCTC	1000
SIL176	TGGAAACCAATGGATCGCTACAGTACTGAAGGGCATGCGAAGATCATCTC	1000
Guichao 2	CTACTTCACTGAAGATGACTCTTCGATCGATCAGAGAAGGTCGGAAGCAG	1050
SIL176	CTACTTCACTGAAGATGACTCTTCGATCGATCAGAGAAGGTCGGAAGCAG	1050
Guichao 2	AGCCTGCCGGAATGTTTGAAGAAGGAATTCCGACTTACAATGAACACTCT	1100
SIL176	AGCCTGCCGGAATGTTTGAAGAAGGAATTCCGACTTACAATGAACACTCT	1100
Guichao 2	CCGATCTGTAGTTACTGGCGATGTCTATGAGGGAATTAGAGCTCTCAGCA	1150
SIL176	CCGATCTGTAGTTACTGGCGATGTCTATGAGGGAATTAGAGCTCTCAGCA	1150
Guichao 2	TCGACAAAGACAATGCCCCTAAGTGGAGTCCTGCTACCCTTGAG---GTC	1197
SIL176	TCGACAAAGACAATGCCCCTAAGTGGAGTCCTGCTACCCTTGAGGAGGTC	1200
Guichao 2	AAGAACGAGGACATCGACCGTCTTTTCGAACCATTCAGTTCAGAAAAGGA	1247
SIL176	AAGAACGAGGACATCGACCGTCTTTTCGAACCATTCAGTTCAGAAAAGGA	1250
Guichao 2	GCTCCAAGTCCCATCTGACGATTCCAACAGATGGAGTGGCAAATTTGAGC	1297
SIL176	GCTCCAAGTCCCATCTGACGATTCCAACAGATGGAGTGGCAAATTTGAGC	1300
Guichao 2	ACACAGTCTATGGCAGAACTTCAGAGTAATTGGCAGCAGAAATAGATGTT	1347
SIL176	ACACAGTCTATGGCAGAACTTCAGAGTAATTGGCAGCAGAAATAGATGTT	1350
Guichao 2	CATCATCCTCACAAGCATGTTTTAAGTACTCGTAACCCCATGTATTCTCA	1397
SIL176	CATCATCCTCACAAGCATGTTTTAAGTACTCGTAACCCCATGTATTCTCA	1400

Guichao 2	TACTTCCCTGTATAACATTGTAACACAAAAAATTGAAAATTACATGACGC	1447
SIL176	TACTTCCCTGTATAACATTGTAACACAAAAAATTGAAAATTACATGACGC	1450
Guichao 2	ACGAAGCAATAAAGCATCTCAAATGTTTTGTT	1479
SIL176	ACGAAGCAATAAAGCATCTCAAATGTTTTGTT	1482

d

Guichao 2	MAEPEQQQQQANPDEVVLGQETGGARVAILNRPRQLNVISDRVVYLLAQF	50
SIL176	MAEPEQQQQQANPDEVVLGQETGGARVAILNRPRQLNVISDRVVYLLAQF	50
Guichao 2	LESWEKDEDAKLVIFKGAGRAFSAGGDLKMFYEGKSDDSCLEVVYRMYWL	100
SIL176	LESWEKDEDAKLVIFKGAGRAFSAGGDLKMFYEGKSDDSCLEVVYRMYWL	100
Guichao 2	CYHIHTYKKTAVALVNGLVMGGGAAMVAPLKFAVVTEKTVFATPEASVGL	150
SIL176	CYHIHTYKKTAVALVNGLVMGGGAAMVAPLKFAVVTEKTVFATPEASVGL	150
Guichao 2	HTDCSFSYIHSRLPGYLGEYLALTGARLNAKEMIAAGLATHFVPSEKLEE	200
SIL176	HTDCSFSYIHSRLPGYLGEYLALTGARLNAKEMIAAGLATHFVPSEKLEE	200
Guichao 2	LEKCLLNLNTGDESAVRAAIEEFSTDVQPDEDSILNKLPTINKCFSAETI	250
SIL176	LEKCLLNLNTGDESAVRAAIEEFSTDVQPDEDSILNKLPTINKCFSAETI	250
Guichao 2	EDIIKAFESEGSIDGNQWIATVLKGMRRSSPTSLKMTLRSIREGRKQSLP	300
SIL176	EDIIKAFESEGSIDGNQWIATVLKGMRRSSPTSLKMTLRSIREGRKQSLP	300
Guichao 2	ECLKKEFRLTMNTLRSVVTGDVYEGIRALSIDKDNAPKWSPATL-EVKNE	349
SIL176	ECLKKEFRLTMNTLRSVVTGDVYEGIRALSIDKDNAPKWSPATLEEVKNE	350
Guichao 2	DIDRLFEPFSSEKELQVPSDDSNRWSGKFEHTVYGRTSE	388
SIL176	DIDRLFEPFSSEKELQVPSDDSNRWSGKFEHTVYGRTSE	389

Supplementary Figure 3 | Sequence of *NOG1* in Guichao 2 and SIL176.

(a) Comparison of 4.3-kb mapping region between Guichao 2 and SIL176. The 12-bp InDel, 15 SNPs, and Transcription Start Sites (TSS) are highlighted.

(b) Full length cDNA of *NOG1* and predicted amino acid sequence. The lowercase letters in

the nucleotide sequence respectively indicate 5'-UTR and 3'-UTR.

(c) Comparison of full length cDNA of *NOG1* between Guichao 2 and SIL176. The 3-bp insertion in SIL176 is highlighted in red.

(d) Comparison of amino acid sequence of *NOG1* between Guichao 2 and SIL176. The amino acid insertion in SIL176 is highlighted in red.

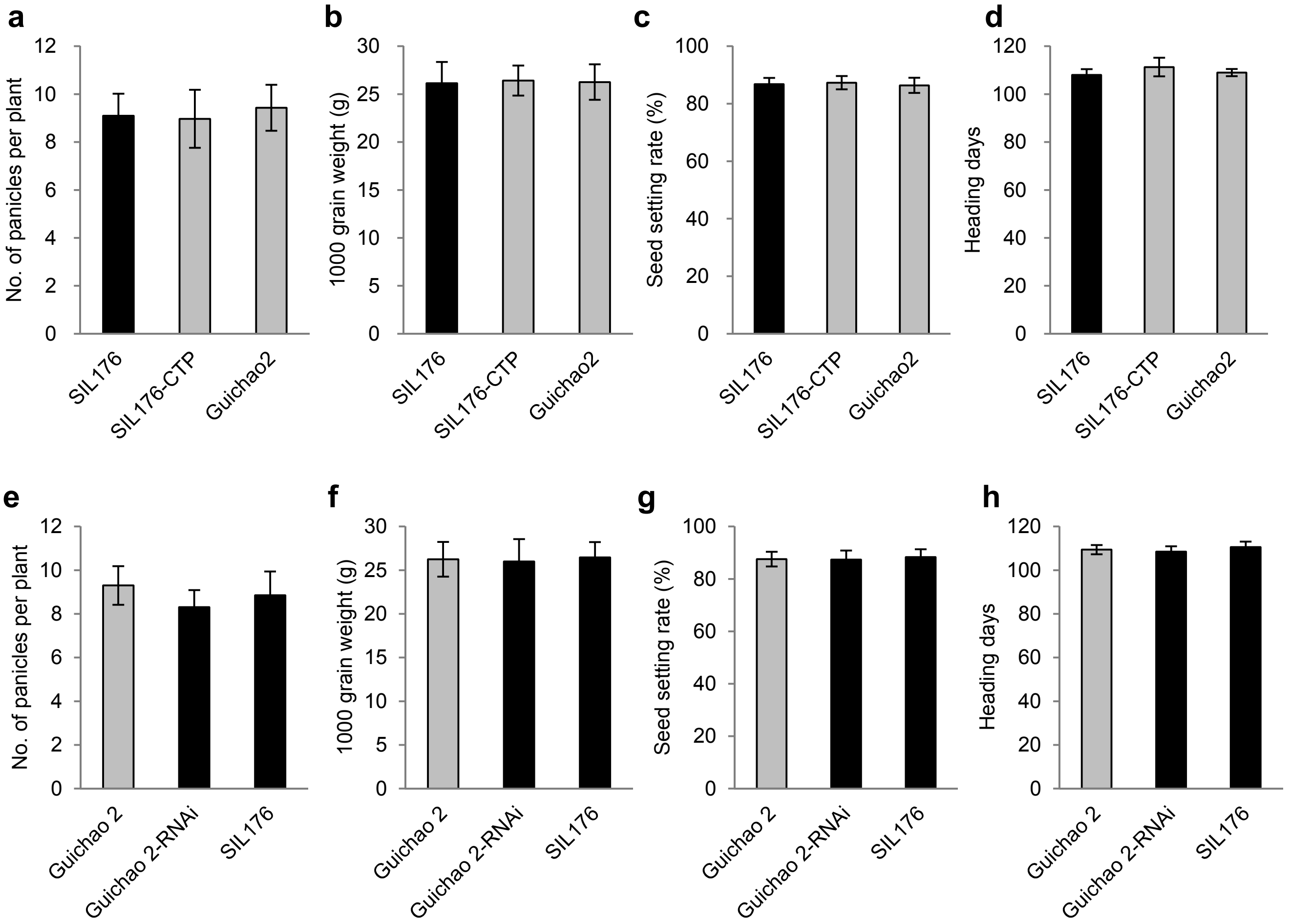

Supplementary Figure 4 | Comparison of other traits among parental and transgenic plants.

(a-d) Comparison of number of panicles per plant, 1000 grain weight, seed setting rate, and heading date among SIL176, SIL176-CTP, and Guichao 2.

(e-h) Comparison of number of panicles per plant, 1000 grain weight, seed setting rate, and heading date among Guichao 2, Guichao 2-RNAi, and SIL176.

Data are means, with error bars showing SEM (n=30).

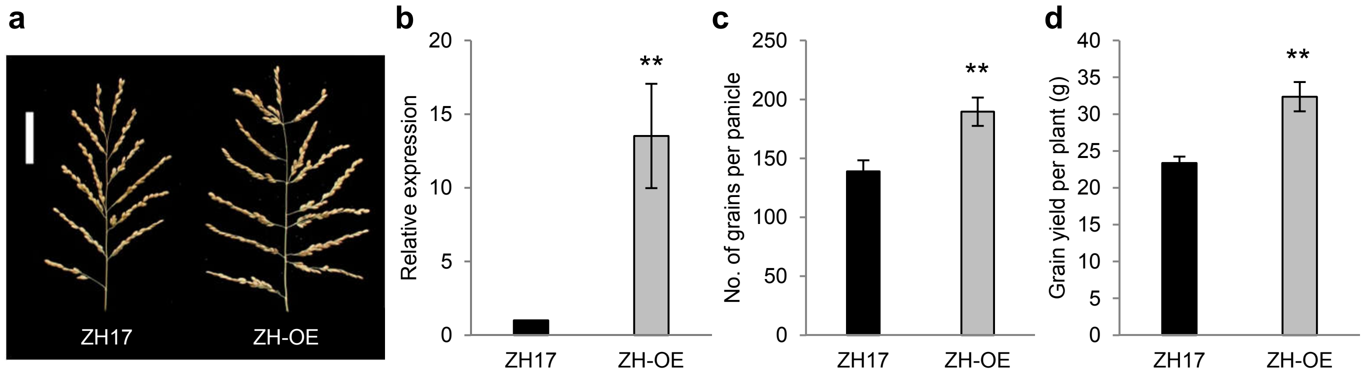

Supplementary Figure 5 | Expression levels of *NOG1* affects grain number and grain yield.

(a) Comparison of main panicle of Zhonghua 17 (ZH17, transgenic control) and *nog1* overexpression transgenic plants (ZH-OE). Scale bars, 5cm.

(b) Comparison of *NOG1* expression levels between ZH17 and ZH-OE (n=4)

(c-d) Comparison of number of grains per panicle and grain yield per plant between ZH17 and ZH-OE (n=30).

Data are means, with error bars showing SEM (two tailed Student's *t* test; **, $P < 0.01$).

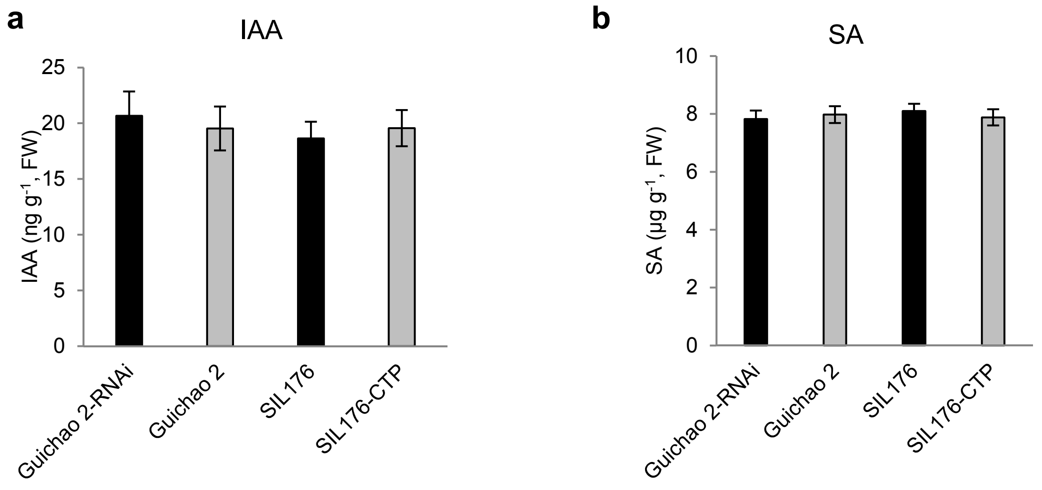

Supplementary Figure 6 | Comparison of IAA and SA levels in young panicles of parental and transgenic plants.

Data are means, with error bars showing SEM (n=10).

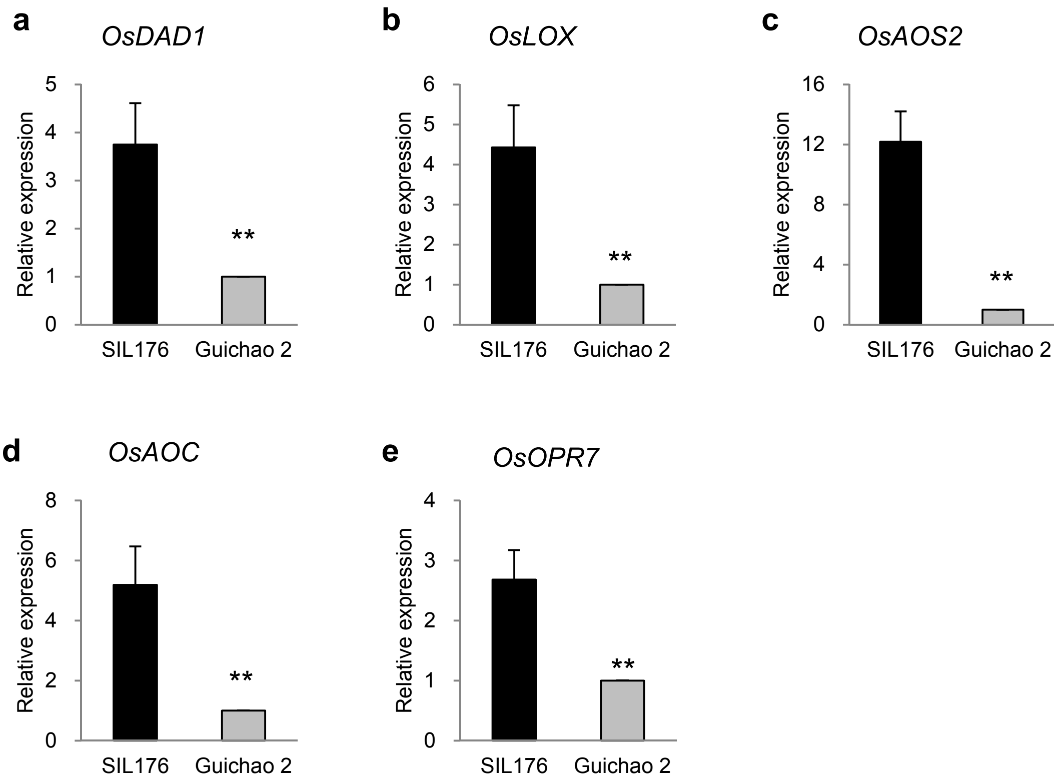

Supplementary Figure 7 | Comparison of expression levels of genes associated with JA pathway between SIL176 and Guichao 2.

Data are means, with error bars showing SEM, n=3 (two-tailed Student's *t* test; **, $P < 0.01$).

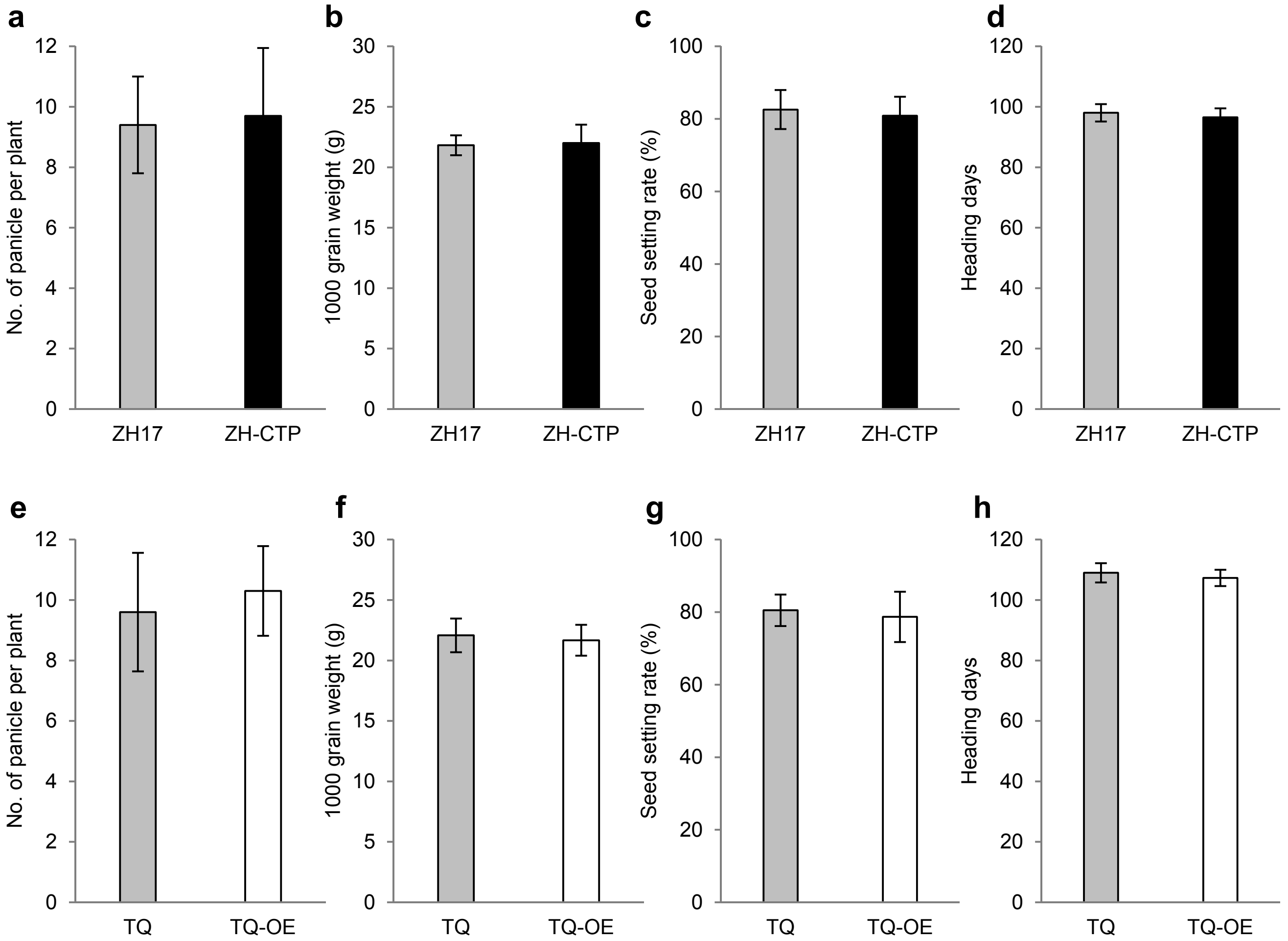

Supplementary Figure 8 | Comparison of other traits in cultivars and transgenic plants.

(a-d) Comparison of number of panicles per plant, 1000 grain weight, seed setting rate, and heading date between Zhonghua 17 and complemented transgenic plants.

(e-h) Comparison of number of panicles per plant, 1000 grain weight, seed setting rate, and heading date between Teqing and overexpression transgenic plants.

Data are means, with error bars showing SEM (n = 30).

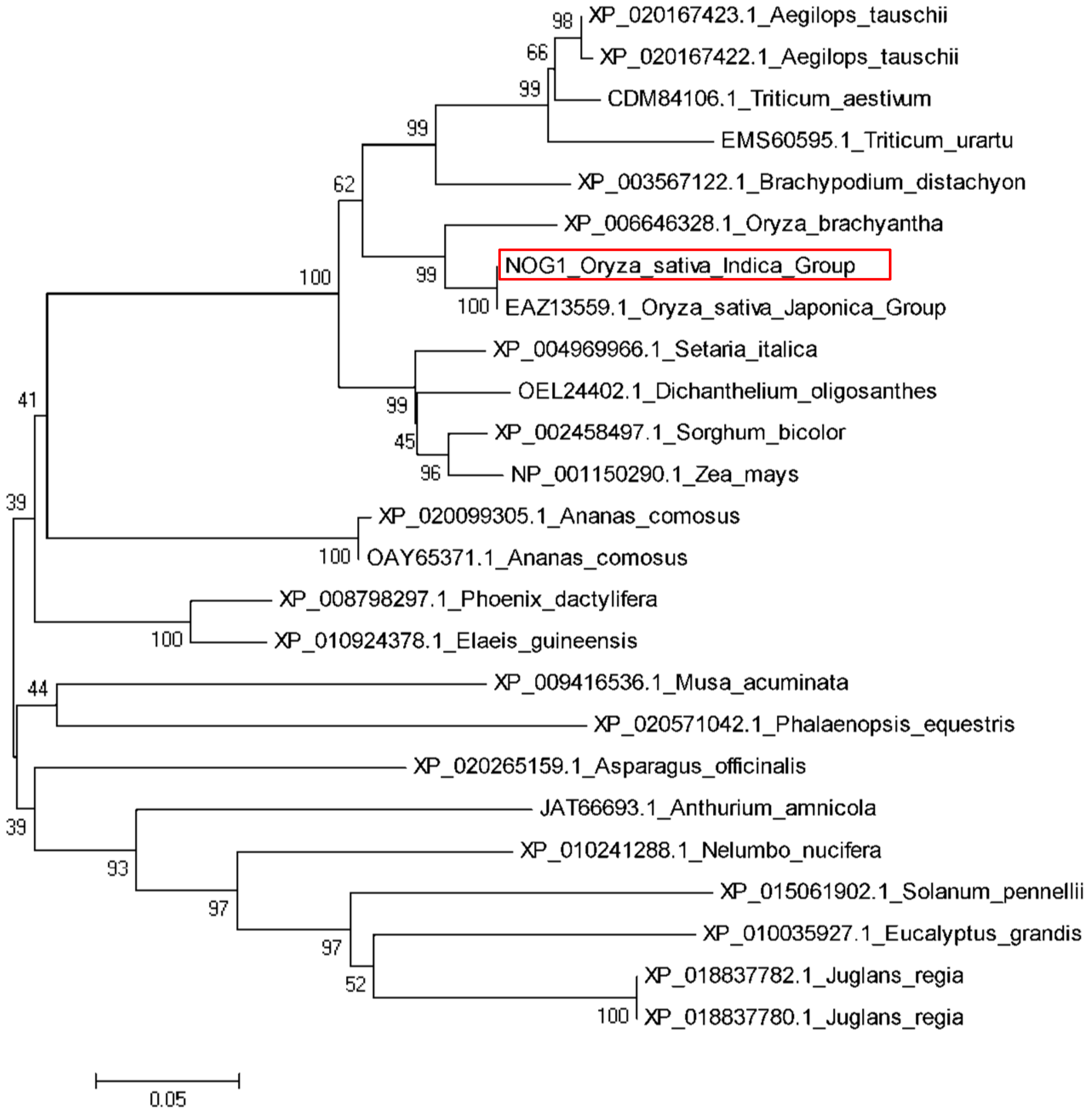

Supplementary Figure 9 | Homology analysis of the NOG1 protein.

Amino acid sequences were aligned using the ClustalX program. The phylogenetic tree was constructed by the software MEGA 5. The evolutionary history was inferred using the Neighbor-Joining method. Test of phylogeny using the bootstrap method, the number of bootstrap replications (1000) are shown next to the branches. Scale bars, the evolutionary distances which were computed using the Jones-Taylor-Thomton (JTT) model.

Supplementary Table 1 | QTL analysis using 243 F_2 individuals.

Phenotype	Locus	LOD	PV (%)	Add	Dom
number of grains per main panicle	RM1183-RM297	7.2	13	17.6	10.8
grain yield per plant	RM1183-RM297	6.8	12	5.2	2.0

QTL was detected by single-point analysis. LOD was performed using the software Map Manager QTX; PV: the phenotypic variance explained by the QTL; Add: additive effects, positive value means Guichao 2 carried alleles increasing trait values; Dom: dominant effect.

Supplementary Table 2 | Fatty acid composition in young panicles of parents and transgenic plants.

Fatty acid composition	Percentage of total						
	Guichao 2-RNAi	Guichao 2		SIL176		SIL176-CTP	
C8:0	0.5±0.02	0.65±0.03	**	0.58±0.04	NS	0.6±0	NS
C10:0	0.51±0.01	0.99±0.04	**	0.81±0.01	*	0.94±0.08	NS
C12:0	0.98±0.03	2.14±0.03	**	2.2±0.14	NS	1.64±0.02	*
C13:0	0.17±0.01	0.34±0	**	0.52±0.04	*	0.22±0.01	**
C14:0	0.57±0.04	0.73±0.03	*	0.84±0.03	NS	0.76±0.08	NS
C15:0	0.27±0.02	0.16±0.01	**	0.2±0	**	0.2±0.01	NS
C15:1	0.95±0.03	0.88±0.03	NS	0.95±0	*	1.07±0	**
C16:0	28.31±0.06	28.06±0.09	NS	25.4±0.09	**	28.49±0.19	**
C16:1	0.87±0	1.34±0	**	0.71±0.01	**	1.01±0.01	**
C17:0	0.31±0.02	0.27±0.01	NS	0.31±0.01	*	0.35±0	**
C18:0	6.63±0.11	5.58±0.19	**	6.61±0.03	**	7.42±0.36	NS
C18:1	8.11±0.35	7.88±0.01	NS	9.89±0.08	**	7.99±0.03	**
C18:2	31.53±0.02	32.48±0.15	**	29.81±0.13	**	30.98±0.11	**
C18:3	13.21±0.31	10.15±0.28	**	13.95±0.07	**	11.15±0.12	**
C20:0	0.84±0.01	0.81±0.04	NS	0.78±0.01	NS	0.91±0.02	**
C20:1	1.68±0.05	2.65±0.02	**	2.08±0.09	**	1.43±0.01	**
C22:0	0.75±0.01	1.32±0.08	**	0.47±0.03	**	0.5±0	NS
C22:1	0.35±0.01	0.32±0	NS	0.76±0.03	**	0.57±0.02	**
C22:6	0.54±0	0.45±0.01	**	0.54±0.01	**	0.66±0	**
C23:0	0.19±0.02	0.24±0.01	NS	0.21±0.01	NS	0.28±0	**
C24:0	2.74±0.06	2.56±0.01	*	2.36±0.04	**	2.82±0.14	*
SUM	100.00	100.00		100.00		100.00	

Data are means, with error bars showing SEM (n=3). Significance showed different from the data in the previous column and was generated by two tailed Student's *t* test, **, $P < 0.01$; *, $P < 0.05$; NS, $P > 0.05$.

Supplementary Table 3 | Rice accessions of cultivated rice in this study.

Number	Name	Origin	Species name	12-bp genotype
ind01	Guichao 2	Guangdong,China	*O.sativa* ssp. *indica*	2 copies of 12-bp
ind02	Minghui 86	Fujian,China	*O.sativa* ssp. *indica*	2 copies of 12-bp
ind03	Teqing	Guangdong,China	*O.sativa* ssp. *indica*	2 copies of 12-bp
ind04	Nov-93	Jiangsu,China	*O.sativa* ssp. *indica*	2 copies of 12-bp
ind05	Liuyenian	Hubei,China	*O.sativa* ssp. *indica*	1 copy of 12-bp
ind06	Hanmadao	Henan,China	*O.sativa* ssp. *indica*	1 copy of 12-bp
ind07	Chaoyang 1B	Zhejiang,China	*O.sativa* ssp. *indica*	2 copies of 12-bp
ind08	JinnanteB	Guangxi,China	*O.sativa* ssp. *indica*	2 copies of 12-bp
ind09	Aijiaonante	Guangdong,China	*O.sativa* ssp. *indica*	2 copies of 12-bp
ind10	Zaoshuxiangheimi	Guangxi,China	*O.sativa* ssp. *indica*	2 copies of 12-bp
ind11	Sanbaili	Hunan,China	*O.sativa* ssp. *indica*	2 copies of 12-bp
ind12	Mamagu	Sichuan,China	*O.sativa* ssp. *indica*	1 copy of 12-bp
ind13	Honggu	Sichuan,China	*O.sativa* ssp. *indica*	1 copy of 12-bp
ind14	Dianrui 409B	Yunnan,China	*O.sativa* ssp. *indica*	2 copies of 12-bp
ind15	04P-182	Zhejiang,China	*O.sativa* ssp. *indica*	2 copies of 12-bp
ind16	Gu 154	Cuba	*O.sativa* ssp. *indica*	2 copies of 12-bp
ind17	Gu 630	Guyana	*O.sativa* ssp. *indica*	2 copies of 12-bp
ind18	Yangdao 2	Jiangsu,China	*O.sativa* ssp. *indica*	2 copies of 12-bp
ind19	Colombo	India	*O.sativa* ssp. *indica*	1 copy of 12-bp
ind20	Mollika(sel)	Nepal	*O.sativa* ssp. *indica*	2 copies of 12-bp
ind21	Lili 356	Korea	*O.sativa* ssp. *indica*	2 copies of 12-bp
ind22	O-Goo-Thou	Nepal	*O.sativa* ssp. *indica*	2 copies of 12-bp
ind23	Sein Ta lay	Myanmar	*O.sativa* ssp. *indica*	1 copy of 12-bp
ind24	Horomamawee	Sri Lanka	*O.sativa* ssp. *indica*	2 copies of 12-bp
ind25	Naw Sadui	Thailand	*O.sativa* ssp. *indica*	2 copies of 12-bp
ind26	XX-4	Thailand	*O.sativa* ssp. *indica*	1 copy of 12-bp
ind27	Chiem Bua	Vietnam	*O.sativa* ssp. *indica*	1 copy of 12-bp
ind28	Re Them Ha Dong	Vietnam	*O.sativa* ssp. *indica*	2 copies of 12-bp
ind29	Beok Montor	Indonesia	*O.sativa* ssp. *indica*	1 copy of 12-bp
ind30	Hr 33	Philippines	*O.sativa* ssp. *indica*	1 copy of 12-bp
ind31	R2389	Philippines	*O.sativa* ssp. *indica*	2 copies of 12-bp
ind32	Aclt	Philippines	*O.sativa* ssp. *indica*	2 copies of 12-bp
ind33	Lchsannde	Philippines	*O.sativa* ssp. *indica*	1 copy of 12-bp
ind34	Agbede	Philippines	*O.sativa* ssp. *indica*	1 copy of 12-bp
ind35	DM 36	Vietnam	*O.sativa* ssp. *indica*	2 copies of 12-bp
ind36	KK15-37-C	Nepal	*O.sativa* ssp. *indica*	1 copy of 12-bp
ind37	Geluo 4	Thailand	*O.sativa* ssp. *indica*	2 copies of 12-bp
ind38	Barkat	India	*O.sativa* ssp. *indica*	1 copy of 12-bp
ind39	NR10045-20-3-2	Nepal	*O.sativa* ssp. *indica*	2 copies of 12-bp
ind40	Mab Suri	Malaysia	*O.sativa* ssp. *indica*	1 copy of 12-bp
ind41	Polayal upland	Sri Lanka	*O.sativa* ssp. *indica*	1 copy of 12-bp
ind42	Tcanuto	Philippines	*O.sativa* ssp. *indica*	1 copy of 12-bp
ind43	Basmati Hunar	Pakistan	*O.sativa* ssp. *indica*	1 copy of 12-bp
ind44	Lateefy	Pakistan	*O.sativa* ssp. *indica*	2 copies of 12-bp
ind45	Tep 2	Vietnam	*O.sativa* ssp. *indica*	1 copy of 12-bp
ind46	DR 32	Vietnam	*O.sativa* ssp. *indica*	2 copies of 12-bp
ind47	Chakula	Bengal	*O.sativa* ssp. *indica*	1 copy of 12-bp
ind48	GH 319	Indonesia	*O.sativa* ssp. *indica*	2 copies of 12-bp
ind49	Muda	Malaysia	*O.sativa* ssp. *indica*	2 copies of 12-bp
ind50	Asebolonkamandi	Indonesia	*O.sativa* ssp. *indica*	1 copy of 12-bp
ind51	Beri	Bengal	*O.sativa* ssp. *indica*	1 copy of 12-bp
ind52	Kathemax	Indonesia	*O.sativa* ssp. *indica*	1 copy of 12-bp
ind53	Jamuna	India	*O.sativa* ssp. *indica*	2 copies of 12-bp
ind54	Kele	Bengal	*O.sativa* ssp. *indica*	1 copy of 12-bp

ind55	Nanxiongzaoyouzhan	Guangdong,China	*O.sativa* ssp. *indica*	2 copies of 12-bp
ind56	Xiangaizao 10	Hunan,China	*O.sativa* ssp. *indica*	2 copies of 12-bp
ind57	Nantehao	Jiangxi, China	*O.sativa* ssp. *indica*	2 copies of 12-bp
ind58	Zaoxian 140	Anhui, China	*O.sativa* ssp. *indica*	2 copies of 12-bp
ind59	Taishannuo	Jiangxi, China	*O.sativa* ssp. *indica*	2 copies of 12-bp
ind60	Hengxianliangchunben	Guangxi,China	*O.sativa* ssp. *indica*	1 copy of 12-bp
ind61	Xuanenchangtanqingzhan	Hubei,China	*O.sativa* ssp. *indica*	1 copy of 12-bp
ind62	Meihuanuo	Sichuan,China	*O.sativa* ssp. *indica*	1 copy of 12-bp
ind63	L301B	Hunan,China	*O.sativa* ssp. *indica*	2 copies of 12-bp
ind64	80B	Hunan,China	*O.sativa* ssp. *indica*	2 copies of 12-bp
ind65	Wanlixian	Hunan,China	*O.sativa* ssp. *indica*	1 copy of 12-bp
ind66	IR661-1	Hunan,China	*O.sativa* ssp. *indica*	2 copies of 12-bp
ind67	Fanhaopi	Yunnan,China	*O.sativa* ssp. *indica*	1 copy of 12-bp
ind68	Jiangnongzao 1B	Jiangxi, China	*O.sativa* ssp. *indica*	2 copies of 12-bp
ind69	Xianghui 91269	Hunan,China	*O.sativa* ssp. *indica*	2 copies of 12-bp
ind70	Aituogu 151	Sichuan,China	*O.sativa* ssp. *indica*	2 copies of 12-bp
ind71	Huangsiguizhan	Guangdong,China	*O.sativa* ssp. *indica*	2 copies of 12-bp
ind72	Chengnongshuijing	Sichuan,China	*O.sativa* ssp. *indica*	2 copies of 12-bp
ind73	Jinxibai	Jiangxi, China	*O.sativa* ssp. *indica*	1 copy of 12-bp
ind74	Qitougu	Yunnan,China	*O.sativa* ssp. *indica*	1 copy of 12-bp
ind75	Ximaxian	Yunnan,China	*O.sativa* ssp. *indica*	1 copy of 12-bp
ind76	Wuzuihonggu	Yunnan,China	*O.sativa* ssp. *indica*	1 copy of 12-bp
ind77	Maweizhan	Guizhou,China	*O.sativa* ssp. *indica*	1 copy of 12-bp
ind78	Taizhong 65	Taiwan,China	*O.sativa* ssp. *indica*	2 copies of 12-bp
ind79	Menjiading 2	Hainan,China	*O.sativa* ssp. *indica*	2 copies of 12-bp
ind80	Qingsiai 16B	Guangdong,China	*O.sativa* ssp. *indica*	1 copy of 12-bp
ind81	Zhenxian 232	Jiangsu,China	*O.sativa* ssp. *indica*	2 copies of 12-bp
ind82	Aimi	Jiangxi, China	*O.sativa* ssp. *indica*	1 copy of 12-bp
ind83	Jinyou 1	Fujian,China	*O.sativa* ssp. *indica*	2 copies of 12-bp
ind84	Xiangzaoxian 7	Hunan,China	*O.sativa* ssp. *indica*	2 copies of 12-bp
jap01	Nipponbare	Japan	*O.sativa* ssp. *japonica*	1 copy of 12-bp
jap02	Zhonghua 17	Beijing,China	*O.sativa* ssp. *japonica*	1 copy of 12-bp
jap03	C418	Liaoning,China	*O.sativa* ssp. *japonica*	1 copy of 12-bp
jap04	Xingguo	Jilin,China	*O.sativa* ssp. *japonica*	1 copy of 12-bp
jap05	Longhuamaohulu	Hebei,China	*O.sativa* ssp. *japonica*	1 copy of 12-bp
jap06	Shuiyuan300li	Tianjin,China	*O.sativa* ssp. *japonica*	1 copy of 12-bp
jap07	Zhonglou1	Shanxi,China	*O.sativa* ssp. *japonica*	1 copy of 12-bp
jap08	Tieganwu	Zhejiang,China	*O.sativa* ssp. *japonica*	2 copies of 12-bp
jap09	Ninghui 21	Jiangsu,China	*O.sativa* ssp. *japonica*	2 copies of 12-bp
jap10	Guihuahuang	Jiangsu,China	*O.sativa* ssp. *japonica*	2 copies of 12-bp
jap11	Jindao 1	Shanxi,China	*O.sativa* ssp. *japonica*	1 copy of 12-bp
jap12	Xugunuo	Hunan,China	*O.sativa* ssp. *japonica*	2 copies of 12-bp
jap13	Jixuenuo	Yunnan,China	*O.sativa* ssp. *japonica*	1 copy of 12-bp
jap14	Nantiangangjiugu	Sichuan,China	*O.sativa* ssp. *japonica*	1 copy of 12-bp
jap15	Gaoyangdiandaodahongmang	Hebei,China	*O.sativa* ssp. *japonica*	1 copy of 12-bp
jap16	Muxiqiu	Shanghai,China	*O.sativa* ssp. *japonica*	1 copy of 12-bp
jap17	Youmangzaojing	Shanghai,China	*O.sativa* ssp. *japonica*	1 copy of 12-bp
jap18	Feidongtangdao	Anhui,China	*O.sativa* ssp. *japonica*	1 copy of 12-bp
jap19	Chiqiaonuo	Fujian,China	*O.sativa* ssp. *japonica*	1 copy of 12-bp
jap20	Bawangbian 1	Hubei,China	*O.sativa* ssp. *japonica*	2 copies of 12-bp
jap21	Xibainian	Sichuan,China	*O.sativa* ssp. *japonica*	1 copy of 12-bp
jap22	LimingB	Liaoning,China	*O.sativa* ssp. *japonica*	1 copy of 12-bp
jap23	Younian	Guizhou,China	*O.sativa* ssp. *japonica*	1 copy of 12-bp
jap24	Do Khao	Laos	*O.sativa* ssp. *japonica*	1 copy of 12-bp
jap25	Padi Lasat	Malaysia	*O.sativa* ssp. *japonica*	1 copy of 12-bp
jap26	Fukuyama	Japan	*O.sativa* ssp. *japonica*	1 copy of 12-bp
jap27	System 400	Korea	*O.sativa* ssp. *japonica*	1 copy of 12-bp

jap28	Sipapak	Indonesia	*O.sativa* ssp. *japonica*	2 copies of 12-bp
jap29	Libu	Philippines	*O.sativa* ssp. *japonica*	1 copy of 12-bp
jap30	Kutube	Philippines	*O.sativa* ssp. *japonica*	1 copy of 12-bp
jap31	Bunian	Philippines	*O.sativa* ssp. *japonica*	1 copy of 12-bp
jap32	Mackkouk	Laos	*O.sativa* ssp. *japonica*	1 copy of 12-bp
jap33	Methinoi	Laos	*O.sativa* ssp. *japonica*	1 copy of 12-bp
jap34	Chablichabolglang	Thailand	*O.sativa* ssp. *japonica*	1 copy of 12-bp
jap35	Kenteng	Laos	*O.sativa* ssp. *japonica*	1 copy of 12-bp
jap36	Lepsang	Laos	*O.sativa* ssp. *japonica*	1 copy of 12-bp
jap37	Jabonl	Bengal	*O.sativa* ssp. *japonica*	1 copy of 12-bp
jap38	Qingfeng	Japan	*O.sativa* ssp. *japonica*	1 copy of 12-bp
jap39	Balsamo	Philippines	*O.sativa* ssp. *japonica*	2 copies of 12-bp
jap40	Rathu Heenati	Sri Lanka	*O.sativa* ssp. *japonica*	1 copy of 12-bp
jap41	Pingrang 10	Korea	*O.sativa* ssp. *japonica*	1 copy of 12-bp
jap42	Dupanyui	Indonesia	*O.sativa* ssp. *japonica*	1 copy of 12-bp
jap43	Pingbei 5	Korea	*O.sativa* ssp. *japonica*	1 copy of 12-bp
jap44	Gaoliqiu	Korea	*O.sativa* ssp. *japonica*	1 copy of 12-bp
jap45	Milizu	Korea	*O.sativa* ssp. *japonica*	1 copy of 12-bp
jap46	Changzhong	Jiangsu,China	*O.sativa* ssp. *japonica*	1 copy of 12-bp
jap47	Sanzhaoqi	Jiangsu,China	*O.sativa* ssp. *japonica*	1 copy of 12-bp
jap48	Huangdao	Jiangsu,China	*O.sativa* ssp. *japonica*	1 copy of 12-bp
jap49	Putaolvzhong	Shanghai,China	*O.sativa* ssp. *japonica*	1 copy of 12-bp
jap50	Muxiuqiu	Shanghai,China	*O.sativa* ssp. *japonica*	2 copies of 12-bp
jap51	Chiheitouhong	Zhejiang,China	*O.sativa* ssp. *japonica*	1 copy of 12-bp
jap52	Chenjianuo	Jiangsu,China	*O.sativa* ssp. *japonica*	1 copy of 12-bp
jap53	Baimaodao	Heilongjiang,China	*O.sativa* ssp. *japonica*	1 copy of 12-bp
jap54	Yelicanghua	Hebei,China	*O.sativa* ssp. *japonica*	1 copy of 12-bp
jap55	Weiguo	Liaoning,China	*O.sativa* ssp. *japonica*	1 copy of 12-bp
jap56	Qitoubaigu	Yunnan,China	*O.sativa* ssp. *japonica*	2 copies of 12-bp
jap57	AnnongwanjingB	Hunan,China	*O.sativa* ssp. *japonica*	2 copies of 12-bp
jap58	76-1	Liaoning,China	*O.sativa* ssp. *japonica*	1 copy of 12-bp
jap59	Zhonghua8	Beijing,China	*O.sativa* ssp. *japonica*	1 copy of 12-bp
jap60	Hongqi 5	Hunan,China	*O.sativa* ssp. *japonica*	1 copy of 12-bp
jap61	Lengshuigu 2	Yunnan,China	*O.sativa* ssp. *japonica*	1 copy of 12-bp
jap62	Dangyu 5	Anhui, China	*O.sativa* ssp. *japonica*	2 copies of 12-bp
jap63	Guangkexiangnuo	Guangxi,China	*O.sativa* ssp. *japonica*	1 copy of 12-bp
jap64	Xiaohonggu	Yunnan,China	*O.sativa* ssp. *japonica*	1 copy of 12-bp
jap65	Wuzidui	Yunnan,China	*O.sativa* ssp. *japonica*	1 copy of 12-bp
jap66	Banjiemang	Yunnan,China	*O.sativa* ssp. *japonica*	1 copy of 12-bp
jap67	Magunuo	Guizhou,China	*O.sativa* ssp. *japonica*	1 copy of 12-bp
jap68	Hongkezhenuo	Guizhou,China	*O.sativa* ssp. *japonica*	1 copy of 12-bp
jap69	Menjiagao 1	Hainan,China	*O.sativa* ssp. *japonica*	2 copies of 12-bp
jap70	Peic 122	Hunan,China	*O.sativa* ssp. *japonica*	2 copies of 12-bp
jap71	Lixinjing	Sichuan,China	*O.sativa* ssp. *japonica*	1 copy of 12-bp
jap72	Chenwan 3	Hunan,China	*O.sativa* ssp. *japonica*	1 copy of 12-bp
jap73	Zimangfeienuo	Guizhou,China	*O.sativa* ssp. *japonica*	1 copy of 12-bp
jap74	Taizhonghr 539	Taiwan,China	*O.sativa* ssp. *japonica*	1 copy of 12-bp

Supplementary Table 4 | Rice accessions of wild rice in this study.

Number	Name	Origin	Species name	12-bp genotype
ruf01	C4	Yuanjiang,China	*O. rufipogon*	12-bp
ruf02	94W1	Dongxiang,China	*O. rufipogon*	12-bp
ruf03	W1944	Chaling,China	*O. rufipogon*	12-bp
ruf04	YD6-0128	Chaling,China	*O. rufipogon*	12-bp
ruf05	S6233	Guangdong,China	*O. rufipogon*	12-bp
ruf06	YD2-0344	Guangxi,China	*O. rufipogon*	12-bp
ruf07	YD2-0772	Guangxi,China	*O. rufipogon*	12-bp
ruf08	YD2-0975	Guangxi,China	*O. rufipogon*	12-bp
ruf09	YD2-0775	Guangxi,China	*O. rufipogon*	12-bp
ruf10	YD4-176	Jiangxi,China	*O. rufipogon*	12-bp
ruf11	12HW14	Nigeria	*O.bathii*	12-bp
ruf12	12HW43	Dongxiang,China	*O. rufipogon*	12-bp
ruf13	12HW20	Ethiopia	*O.longistaminata*	12-bp
ruf14	YD6-0001	Chaling,China	*O.rufipogon*	12-bp
ruf15	IRGC100599	Taiwan,China	*O.rufipogon*	12-bp
ruf16	05BW17	Guangdong,China	*O.officinalis*	12-bp
ruf17	1-63	Yunnan,China	*O. rufipogon*	12-bp
ruf18	TB2132	Gaozhou,China	*O.rufipogon*	12-bp
ruf19	TB2010	India	*O.nivara*	12-bp
ruf20	14WR1	Jiangxi,China	*O. rufipogon*	12-bp
ruf21	14WR4	Guangxi,China	*O. rufipogon*	12-bp
ruf22	14WR7	Guangxi,China	*O. rufipogon*	12-bp
ruf23	14WR10	Guangxi,China	*O. rufipogon*	12-bp
ruf24	14WR12	Guangxi,China	*O. rufipogon*	12-bp
ruf25	14WR13	Guangxi,China	*O. rufipogon*	12-bp
ruf26	14WR14	Guangxi,China	*O. rufipogon*	12-bp
ruf27	14WR17	Guangxi,China	*O. rufipogon*	12-bp
ruf28	14WR19	Guangxi,China	*O. rufipogon*	12-bp
ruf29	14WR21	Hainan,China	*O. rufipogon*	12-bp
ruf30	14WR24	Hainan,China	*O. rufipogon*	12-bp
ruf31	14WR25	Hainan,China	*O. rufipogon*	12-bp
ruf32	14WR27	Hainan,China	*O. rufipogon*	12-bp
ruf33	14WR28	Hainan,China	*O. rufipogon*	12-bp
ruf34	14WR30	Hainan,China	*O. rufipogon*	12-bp
ruf35	14WR32	Guangdong,China	*O. rufipogon*	12-bp
ruf36	14WR35	Guangdong,China	*O. rufipogon*	12-bp
ruf37	14WR36	Guangdong,China	*O. rufipogon*	12-bp
ruf38	14WR38	Jiangxi,China	*O. rufipogon*	12-bp
ruf39	14WR42	Hunan,China	*O. rufipogon*	12-bp
ruf40	14WR47	Cambodia	*Oryza* sp.	12-bp
ruf41	14WR48	Cambodia	*Oryza* sp.	12-bp
ruf42	14WR49	Cambodia	*Oryza* sp.	12-bp
ruf43	14WR50	Laos	*O.spontanea*	12-bp
ruf44	14WR51	Laos	*O. rufipogon*	12-bp
ruf45	14WR53	Bangladesh	*O. rufipogon*	12-bp
ruf46	14WR54	Bangladesh	*O. rufipogon*	12-bp
ruf47	14WR56	Laos	*O.nivara*	12-bp
ruf48	14WR57	Laos	*O.nivara*	12-bp
ruf49	14WR61	Laos	*O.nivara*	12-bp
ruf50	14WR63	Laos	*O. rufipogon*	12-bp

ruf51	14WR64	Laos	*O. rufipogon*	12-bp
ruf52	14WR65	Cambodia	*O.nivara*	12-bp
ruf53	14WR66	Cambodia	*O.nivara*	12-bp
ruf54	14WR67	Cambodia	*O. rufipogon*	12-bp
ruf55	14WR69	Cambodia	*O. rufipogon*	12-bp
ruf56	14WR79	Taiwan,China	*O.nivara*	12-bp
ruf57	14WR80	Philippines	*O. rufipogon*	12-bp
ruf58	14WR82	Thailand	*Oryza* sp.	12-bp
ruf59	14WR83	Thailand	*Oryza* sp.	12-bp
ruf60	14WR86	Thailand	*O. rufipogon*	12-bp
ruf61	14WR87	Thailand	*O.nivara*	12-bp
ruf62	14WR91	Thailand	*O.nivara*	12-bp
ruf63	14WR93	India	*O. rufipogon*	12-bp
ruf64	14WR99	Sri Lanka	*O.nivara*	12-bp
ruf65	14WR101	Thailand	*O.rufipogon*	12-bp
ruf66	14WR102	Bangladesh	*O.rufipogon*	12-bp
ruf67	14WR104	Thailand	*O.rufipogon*	12-bp
ruf68	14WR106	Bangladesh	*O.nivara*	12-bp
ruf69	14WR107	India	*O.rufipogon*	12-bp
ruf70	14WR108	India	*O.rufipogon*	12-bp
ruf71	14WR109	India	*O.rufipogon*	12-bp
ruf72	14WR110	Laos	*O.rufipogon*	12-bp
ruf73	14WR111	Laos	*O.rufipogon*	12-bp
ruf74	14WR113	Cambodia	*O.rufipogon*	12-bp
ruf75	14WR114	Cambodia	*O.rufipogon*	12-bp

Supplementary Table 5 | Primers used in this study.

Name	Forward primer (5'-3')	Reverse primer (5'-3')
RM128	AGCTTGGGTGATTTCTTGGAAGCG	ACGACGAGGAGTCGCCGTGCAG
RM1183	GGGCACGAATAAAACCAGAG	GGGATGGTCCAATGACAAAG
RM11669	AAACCGTTCCAGGGAGACTGACC	TCGTCTGATCCATCCATCCATCC
RM297	ACAGGGCTATGCAGACACAGTGC	AGCAAGCGAAGGGAAGTGACC
RM543	AGGAAAGGGCCTAGCGTCTCACC	AAGAGAAACACATCGGCGCAAGG
RM302	TCATGTCATCTACCATCACAC	ATGGAGAAGATGGAATACTTGC
DE4	GTTGTCTTCACTATTTCGAC	GGTTAGCAATGTTATTTTGT
DE17	CTATGTGGTTAGGTCCAT	TGAGGAAGCCTAGTATGA
DE28	GAGGAGGTGGAGGAGGAT	TTTACGAGTATGGAGGGAG
W84	GTGCATCACTGGCTCACATT	AGGTCTGCGAGGTTGAGAAA
NL8	CCTGGTATGTGGCTTTGC	GTCCCTTAATGCCCTCTG
IN4	GGGCAAGCTGACTAAGAA	CCTAATAAACCGAGAAGACAAT
860CX5	ACCCATGACAAGTCCATT	TGTATCTCCTCGCCCAGT
870CX2	CAAGGGAATCTGACACGG	CTCTGGGCTACCAACGAG
NOG1-CTP	GAAGATCTCATCTGATGCCTCATACTGA	CCGACGCGTCATGCTTAGGCTGTTGAT
NOG1-RNAi-1	GGACTAGTGGGAGAAAGATGAGGA	TCCGAGCTCGGTCAAAGCCAGGTAC
NOG1-RNAi-2	CGGGATCCGGTCAAAGCCAGGTAC	GGGGTACCAGAGCTGGGAGAAAGA
pGreenII 0800-NOG1	GGGGTACCTGGCACAACCGCACAAAG	CGGGATCCCAGGGAGGGACCCGAATTC
pGreenII 62SK -DOF	GGCTGCAGATGGGGGAGTGCAAGGTG	TTGGTACCTCAAGATCCCTCTTGGAAGGT
UBI-QRT	CTGTCAACTGCCGCAAGAAG	GGCGAGTGACGCTCTAGTTC
NOG1-QRT	TCCGACTTACAATGAACAC	GGTAGCAGGACTCCACTT
870-QRT	GCCAAGTCCAAGTTCTGG	GGTGGTATCACGGTACTCCT
NOG1-GFP	GGGGTACCATGGCGGAGCCGGAGCAGCAG	GCGTCGACCTCTGAAGTTCTGCCATAGACT
In situ hybridization-NOG1	CTTCGCCCAACTCGTTCT	CACCACCTCGTCGGGATT
NOG1-OE	GGGGTACCATGGCGGAGCCGGAGCAGCAG	GGACTAGTTTACTCTGAAGTTCTGCCAT
3' RACE GSP1	GTGGAGTCCTGCTACCCT	
3' RACE GSP2	TGAGGAGGTCAAGAACGA	
5' RACE GSP2	TGGGCGAGGAGATACACC	
5' RACE GSP1	TTGGCATCCTCATCTTTC	
OsDAD1-QRT	ACCTCATCGACGGGTTCAC	CGGTTCACGTACTCCTTCTTCA
OsLOX-QRT	GCATCCCCAACAGCACATC	AATAAAGATTTGGGAGTGACATATTGG
OsAOS2-QRT	CAATACGTGTACTGGTCGAATGG	AAGGTGTCGTACCGGAGGAA
OsAOC-QRT	GAGGCTTCTTGGTAGTAGGTGGA	CGTAGTGGCGGTCGTTGTAGT
OsOPR7-QRT	GGATGTAAATGTACTGCGGGAT	ACAATCTGTGCTGATGACCCA
